# Age‐specific burden of cervical cancer associated with HIV: A global analysis with a focus on sub‐Saharan Africa

**DOI:** 10.1002/ijc.33841

**Published:** 2021-10-19

**Authors:** Ahmadaye Ibrahim Khalil, Tharcisse Mpunga, Feixue Wei, Iacopo Baussano, Catherine de Martel, Freddie Bray, Dominik Stelzle, Scott Dryden‐Peterson, Antoine Jaquet, Marie‐Josèphe Horner, Olutosin A. Awolude, Mario Jesus Trejo, Washington Mudini, Amr S. Soliman, Mazvita Sengayi‐Muchengeti, Anna E. Coghill, Matthys C. van Aardt, Hugo De Vuyst, Stephen E. Hawes, Nathalie Broutet, Shona Dalal, Gary M. Clifford

**Affiliations:** ^1^ Early Detection, Prevention and Infections Branch, International Agency for Research on Cancer (IARC/WHO) Lyon France; ^2^ Butaro Cancer Centre of Excellence, Ministry of Health Butaro Rwanda; ^3^ Cancer Surveillance Branch, International Agency for Research on Cancer (IARC/WHO) Lyon France; ^4^ Center for Global Health, Department of Neurology, Faculty of Medicine Technical University of Munich Munich Germany; ^5^ Chair of Epidemiology, Department of Sport and Health Sciences Technical University of Munich Munich Germany; ^6^ Division of Infectious Diseases Brigham and Women's Hospital Boston Massachusetts USA; ^7^ Department of Immunology and Infectious Diseases Harvard T.H. Chan School of Public Health Boston Massachusetts USA; ^8^ Botswana Harvard AIDS Institute Partnership Gaborone Botswana; ^9^ University of Bordeaux, Inserm, French National Research Institute for Sustainable Development (IRD), UMR 1219 Bordeaux France; ^10^ Infections and Immunoepidemiology Branch, Division of Cancer Epidemiology and Genetics National Cancer Institute Bethesda Maryland USA; ^11^ Department of Obstetrics and Gynaecology, College of Medicine University of Ibadan Ibadan Nigeria; ^12^ Infectious Disease Institute, College of Medicine University of Ibadan Ibadan Nigeria; ^13^ Department of Epidemiology and Biostatistics University of Arizona Tucson Arizona USA; ^14^ Division of Anatomical Pathology, Faculty of Health Sciences University of Cape Town Cape Town South Africa; ^15^ Community Health and Social Medicine Department, CUNY School of Medicine The City College of New York New York City New York USA; ^16^ National Cancer Registry, National Health Laboratory Service Johannesburg South Africa; ^17^ School of Public Health University of the Witwatersrand Johannesburg South Africa; ^18^ South African DSI‐NRF Centre of Excellence in Epidemiological Modelling and Analysis (SACEMA) Stellenbosch University Stellenbosch South Africa; ^19^ Cancer Epidemiology Program, Division of Population Science H. Lee Moffitt Cancer Center and Research Institute Tampa Florida USA; ^20^ Gynaecologic Oncology Unit, Department of Obstetrics and Gynaecology University of Pretoria Pretoria South Africa; ^21^ Departments of Epidemiology, Health Services, and Global Health University of Washington Seattle Washington USA; ^22^ Department of Sexual and Reproductive Health and Research, World Health Organization Geneva Switzerland; ^23^ Department of Global HIV, Hepatitis and STIs Programmes, World Health Organization Geneva Switzerland

**Keywords:** age‐specific incidence rates, cervical cancer, HIV, population‐attributable fraction, sub‐Saharan Africa

## Abstract

HIV substantially worsens human papillomavirus (HPV) carcinogenicity and contributes to an important population excess of cervical cancer, particularly in sub‐Saharan Africa (SSA). We estimated HIV‐ and age‐stratified cervical cancer burden at a country, regional and global level in 2020. Proportions of cervical cancer (a) diagnosed in women living with HIV (WLHIV), and (b) attributable to HIV, were calculated using age‐specific estimates of HIV prevalence (UNAIDS) and relative risk. These proportions were validated against empirical data and applied to age‐specific cervical cancer incidence (GLOBOCAN 2020). HIV was most important in SSA, where 24.9% of cervical cancers were diagnosed in WLHIV, and 20.4% were attributable to HIV (vs 1.3% and 1.1%, respectively, in the rest of the world). In all world regions, contribution of HIV to cervical cancer was far higher in younger women (as seen also in empirical series). For example, in Southern Africa, where more than half of cervical cancers were diagnosed in WLHIV, the HIV‐attributable fraction decreased from 86% in women ≤34 years to only 12% in women ≥55 years. The absolute burden of HIV‐attributable cervical cancer (approximately 28 000 cases globally) also shifted toward younger women: in Southern Africa, 63% of 5341 HIV‐attributable cervical cancer occurred in women <45 years old, compared to only 17% of 6901 non‐HIV‐attributable cervical cancer. Improved quantification of cervical cancer burden by age and HIV status can inform cervical cancer prevention efforts in SSA, including prediction of the impact of WLHIV‐targeted vs general population approaches to cervical screening, and impact of HIV prevention.

## Abstract 2

What's new?

The cancer‐causing potential of human papillomavirus (HPV) is amplified in patients infected with HIV. This is a particular consideration in sub‐Saharan Africa. Here, the authors developed an age‐specific method to determine the global burden of cervical cancer cases attributable to HIV. They found that around 20% of cervical cancer cases in sub‐Saharan Africa were attributable to HIV. Most of these occurred in women under age 45. These data can inform design of cervical cancer prevention programs, particularly in settings hit by a double burden of HPV and HIV.

**Abbreviations**
ASIR: age‐standardized incidence rate
cART: combination antiretroviral therapy
CI: confidence interval
HR HPV: high risk human papillomavirus
LMIC: low‐ and middle‐income countries
OR: odds ratio
PAF: population‐attributable fraction
RR: relative risk
SSA: sub‐Saharan Africa
WHO: World Health Organization
WLHIV: women living with HIV

## 1 INTRODUCTION

Cervical cancer is a major public health problem, representing the fourth most common cancer in women worldwide and accounting for more than 600 000 new cases and 340 000 global deaths in 2020 (GLOBOCAN 2020 database presented in Global Cancer Observatory proposed by the International Agency for Research on Cancer [IARC]: https://gco.iarc.fr/today/home).^1^ However, this burden is unequally distributed, with 9 out of 10 deaths from cervical cancer occurring in low‐ and middle‐income countries (LMIC), and 6 of those in sub‐Saharan Africa (SSA) alone.^1^ This inequality is partly a product of lack of access to cervical cancer screening and cancer treatment, but also of disparate prevalence of risk factors, including high‐risk human papillomavirus (HR HPV) and HIV infection.

Persistent infection with HR HPV types is the underlying cause of all cervical cancer. However, natural history of HR HPV infection is substantially worsened by HIV‐related immunodeficiency, such that women living with HIV (WLHIV) are at elevated cervical cancer risk. In a recent systematic literature review and meta‐analysis, HIV was estimated to increase cervical cancer risk 6‐fold and, in a subsequent modeling exercise, to account for approximately 5% of the global cervical cancer burden.^2^ HIV‐attributable cervical cancer burden is particularly unequally spread, with 85% of cases diagnosed in SSA alone. In Southern Africa, the SSA region most impacted by the HIV epidemic, more than half of all cervical cancer cases in 2018 were estimated to be attributable to HIV.^2^

In 2020, the World Health Organization (WHO) launched a global call to eliminate cervical cancer as a public health problem, for which the main prevention components are HPV vaccination, cervical cancer screening and management of detected disease.^3^ However, in settings doubly hit by both HPV and HIV epidemics, most notably SSA, progress toward the cervical cancer elimination goal will also be influenced by HIV‐focused prevention measures, such as reducing HIV prevalence, early diagnosis of HIV and timely initiation of combination antiretroviral therapy (cART). In these settings, age‐specific estimates of cervical cancer by HIV status are key to informing the design of appropriate cervical cancer control programs (including the extent to which cervical screening should be prioritized and adapted for WLHIV), as well as to predict their impact.

There has been no previous description of the interaction of HIV infection and age on cervical cancer at a population level. Such an effort has been complicated by the changing epidemiology of the HIV epidemic, first in terms of changing HIV prevalence, but even more so by huge fluctuations in life expectancy due to severe co‐mortality from opportunistic infections (ie, decrease in life expectancy in the early phases of the epidemic, followed by a subsequent increase in the era of wider and earlier access to cART). Indeed, age‐specific estimates of HIV‐attributable cervical cancer were considered beyond the scope of the above‐mentioned meta‐analysis and global modeling exercise,^2^ in recognition of the need for a more targeted approach.

To this end, our aim had two parts: first, to develop a methodology to describe the relative contribution of HIV to age‐specific cervical cancer burden globally, with a particular focus on SSA. This approach was based on using most recent age‐specific estimates of HIV prevalence (UNAIDS) and relative risk (RR), accompanied by a widespread collection of empirical data from cervical cancer series of known HIV status, in order to inform and validate the methodology. Then, as a second step, we applied this methodology to worldwide estimates of cervical cancer incidence (GLOBOCAN 2020),^1^ to estimate HIV‐ and age‐stratified cervical cancer burden at a country, regional and global level.

## 2 METHODS

### 2.1 Country‐specific estimates of cervical cancer and HIV prevalence

Age‐specific population denominators, number of cervical cancer cases and cervical cancer incidence (cases per 100 000 person years) were extracted for 185 individual countries/territories described in GLOBOCAN 2020.^1^

HIV prevalence estimates for women aged ≥15 years in 2019 were acquired from UNAIDS for 175 of the 185 GLOBOCAN countries/territories. Age‐specific denominators (population size) and numerators (number of WLHIV) were provided by UNAIDS in 5‐year age groups and were aggregated to produce HIV prevalence estimates for the age groups 15 to 34, 35 to 44, 45 to 54 and ≥55 years (see justification below). For the 10 remaining GLOBOCAN countries/territories, estimates were completed with similarly aggregated age‐specific HIV prevalence for 2019 published by the Institute for Health Metrics and Evaluation (IHME) (http://ghdx.healthdata.org/gbd-results-tool).

### 2.2 Age‐specific RRs for HIV and cervical cancer

A systematic literature review and meta‐analysis had previously identified 24 studies reporting RRs for cervical cancer in HIV infected women.^2^ Of these 24 studies, only one provided RRs by age group.^4^ This IARC‐led case‐control study, including 560 cervical cancer cases and 155 female non‐cancer controls, was the largest identified by the meta‐analysis^2^ and was undertaken in an unscreened population in Rwanda between 2012 and 2016. Of note, cART was already widespread in Rwanda in this period (with growing availability from 2000; 81% of eligible PLHIV in Rwanda were estimated to be receiving cART by 2016).^5^ RRs were reported as odds ratios (ORs) (adjusted for province of residence), separately for the age groups 15 to 34 (OR = 33.8, 95% confidence interval [CI], 9.3‐122.6), 35 to 44 (6.8, 95% CI, 3.6‐13.1), 45 to 54 (4.2, 95% CI, 2.1‐8.3) and ≥55 years (2.4, 95% CI, 0.9‐6.5).

### 2.3 Empirical evidence on HIV prevalence in cervical cancer, by age

The same literature review described above^2^ also identified 19 epidemiological studies reporting HIV status in consecutively diagnosed series of cervical cancer. These studies were all conducted in SSA, and authors were invited to share data on HIV prevalence according to the age groups ≤34, 35 to 44, 45 to 54 and ≥55 years. Relevant data were obtained for 17 series from 13 countries: Botswana,^6^ Côte d'Ivoire,^7^, ^8^ Kenya,^9^, ^10^ Malawi,^11^ Mozambique,^12^ Nigeria,^13^ Rwanda,^4^ Senegal,^14^ South Africa,^10^, ^15^, ^16^ United Republic of Tanzania,^17^ Uganda,^18^ Zambia^19^ and Zimbabwe.^20^ We were unsuccessful in obtaining age‐stratified data from two additional eligible studies from Malawi.^21^, ^22^

### 2.4 Statistical analysis

For each of the 185 GLOBOCAN countries, and separately by age groups ≤34, 35 to 44, 45 to 54 and ≥55 years, we estimated: (a) the fraction of cervical cancer diagnosed among WLHIV (or HIV prevalence in cervical cancer), and (b) the fraction of cervical cancer attributable to HIV (or population‐attributable fraction [PAF]), according to the following formulas^2^:

1. HIV prevalence in cervical cancer = (HIV prevalence × RR)/([1 − HIV prevalence] + [HIV prevalence × RR]).
2. PAF = (HIV prevalence × [RR − 1])/((1 + HIV prevalence × [RR − 1])).

For each country, and by age group, we calculated the number of new cervical cancer cases (a) diagnosed among WLHIV, and (b) attributable to HIV, respectively, by multiplying these two respective fractions by the number of new cervical cancer cases estimated from GLOBOCAN 2020. Country‐specific estimates were aggregated worldwide, according to WHO region and for the WHO African Region (WHO/AFRO), and additionally according to UN sub‐region, that is, Eastern, Western, Central and Southern Africa, referred to collectively here as SSA. Overall (ie, all ages combined), HIV prevalence and PAFs were derived from the aggregate of age‐specific numerators. Finally, these estimates were applied to age‐standardized incidence rates (ASIRs) of cervical cancer, by HIV attribution status, per 100 000 person years for four SSA sub‐regions, as available in GLOBOCAN 2020.

All statistical analyses were conducted using Stata software (Version 14.2) and world maps drawn using QGIS3 software.

## 3 RESULTS

### 3.1 Fractions of invasive cervical cancer diagnosed among WLHIV and attributable to HIV

In 2020, 5.6% of global cervical cancer cases were estimated to be diagnosed among WLHIV. However, this fraction varied substantially according to age, being 15.5% for cervical cancer diagnosed at ≤34 years, 9.4% at 35 to 44, 5.6% at 45 to 54 and 1.7% at ≥55 years (Table 1). The overall fraction of cervical cancer directly attributable to HIV (ie, PAF) was 4.6% globally and was similarly inversely related to age, being 15.1% at ≤34 years, 8.0% at 35 to 44, 4.2% at 45 to 54 and 1.0% at ≥55 years, respectively.

**TABLE 1:** HIV prevalence and HIV‐attributable fraction (PAF) in cervical cancer by age group, WHO region^a^ and sub‐Saharan Africa sub‐region^b^ in 2020

	Age group (years)									
	≤34		35‐44		45‐54		≥55		Overall	
	HIV prevalence^c^ (%)	PAF (%)	HIV prevalence^c^ (%)	PAF (%)	HIV prevalence^c^ (%)	PAF (%)	HIV prevalence^c^ (%)	PAF (%)	HIV prevalence^c^ (%)	PAF (%)
World	15.5	15.1	9.4	8.0	5.6	4.2	1.7	1.0	5.6	4.6
Africa (AFRO)	51.8	50.2	38.6	32.9	27.1	20.6	8.7	5.1	24.9	20.4
Southern Africa	88.5	85.9	80.3	68.5	62.6	47.7	21.1	12.3	53.8	43.5
Eastern Africa	58.5	56.8	44.1	37.6	32.4	24.7	10.7	6.3	28.9	23.4
Central Africa	36.6	35.5	22.5	19.2	12.2	9.3	3.2	1.9	12.1	10.0
Western Africa	27.2	26.4	16.2	13.9	10.5	8.0	3.1	1.8	10.7	8.9
Non‐AFRO regions	4.6	4.5	2.1	1.8	1.1	0.8	0.2	0.1	1.3	1.1
Europe (EURO)	8.4	8.2	3.6	3.1	1.4	1.0	0.3	0.2	2.3	2.0
South‐East Asia (SEARO)	6.1	5.9	2.4	2.1	1.1	0.8	0.3	0.2	1.2	1.0
Americas (PAHO)	5.1	5.0	2.9	2.4	1.9	1.4	0.5	0.3	1.9	1.6
Western Pacific (WPRO)	1.8	1.8	0.9	0.8	0.4	0.3	0.0	0.0	0.5	0.4
East Mediterranean (EMRO)	1.8	1.7	0.9	0.8	0.5	0.4	0.0	0.0	0.5	0.4

Abbreviations: PAF, population‐attributable fraction; SSA, sub‐Saharan Africa; WLHIV, women living with HIV.

^a^ WHO regions include Africa (AFRO), Americas (PAHO), East Mediterranean (EMRO), Europe (EURO), South‐East Asia (SEARO) and Western Pacific (WPRO).

^b^ SSA sub‐regions include Eastern Africa, Western Africa, Central Africa and Southern Africa.

^c^ HIV prevalence in cervical cancer, equivalent to the fraction of cervical cancer diagnosed in WLHIV.

HIV was much more predominant in SSA (WHO/AFRO) (24.9% of cervical cancer diagnosed in WLHIV, 20.4% attributable to HIV) than in other WHO regions (1.3% and 1.1%, respectively), but consistent age‐specific decreases in HIV proportions were seen in all world regions (Table 1). In SSA, estimated HIV prevalence was 51.8% among cervical cancer diagnosed at ≤34 years, 38.6% at 35 to 44, 27.1% at 45 to 54 years and 8.7% among women ≥55 years. The PAF in SSA was 50.2% in women ≤34 years, 32.9% in 35 to 44 years, 20.6% in 45 to 54 years and 5.1% at ≥55 years (Table 1).

Within SSA, the proportion of cervical cancer diagnosed in WLHIV ranged from 53.8% in Southern Africa (PAF = 43.5%) down to 10.7% (PAF = 8.9%) in Western Africa (Table 1). In Southern Africa, estimated HIV positivity was 88.5% among cervical cancer diagnosed at ≤34 years, 80.3% in 35 to 44 years, 62.6% in 45 to 54 years and 21.1% at ≥55 years, while PAFs were 85.9% in women ≤34 years, 68.5% in 35 to 44 years, 47.7% in 45 to 54 years and 12.3% at ≥55 years (Table 1).

**FIGURE 1:**
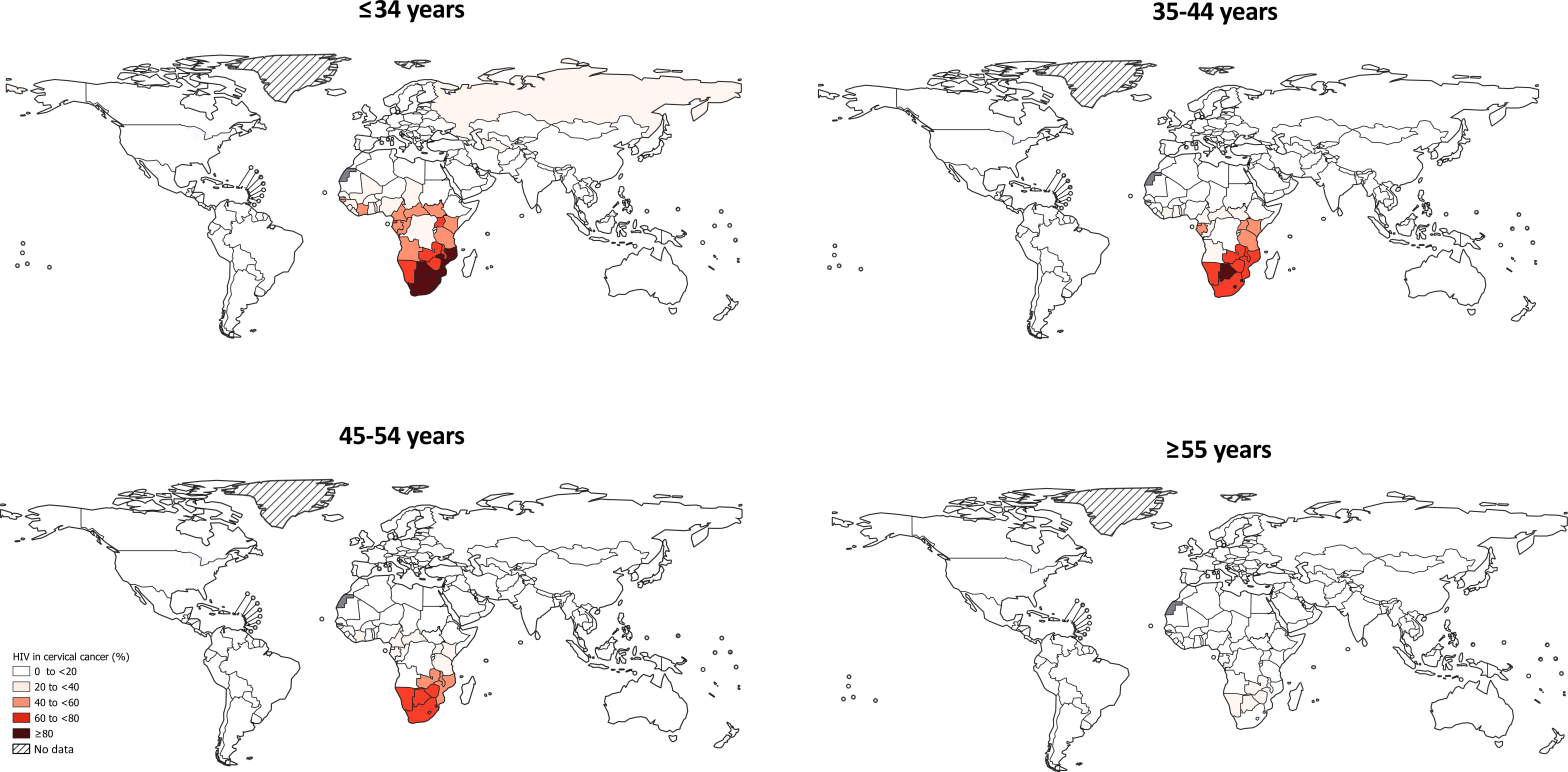
HIV prevalence in cervical cancer in 2020, by age group. The designations used and the presentation of the material in this article do not imply the expression of any opinion whatsoever on the part of WHO and the IARC about the legal status of any country, territory, city, or area, or of its authorities, or concerning the delimitation of its frontiers or boundaries

**FIGURE 2:**
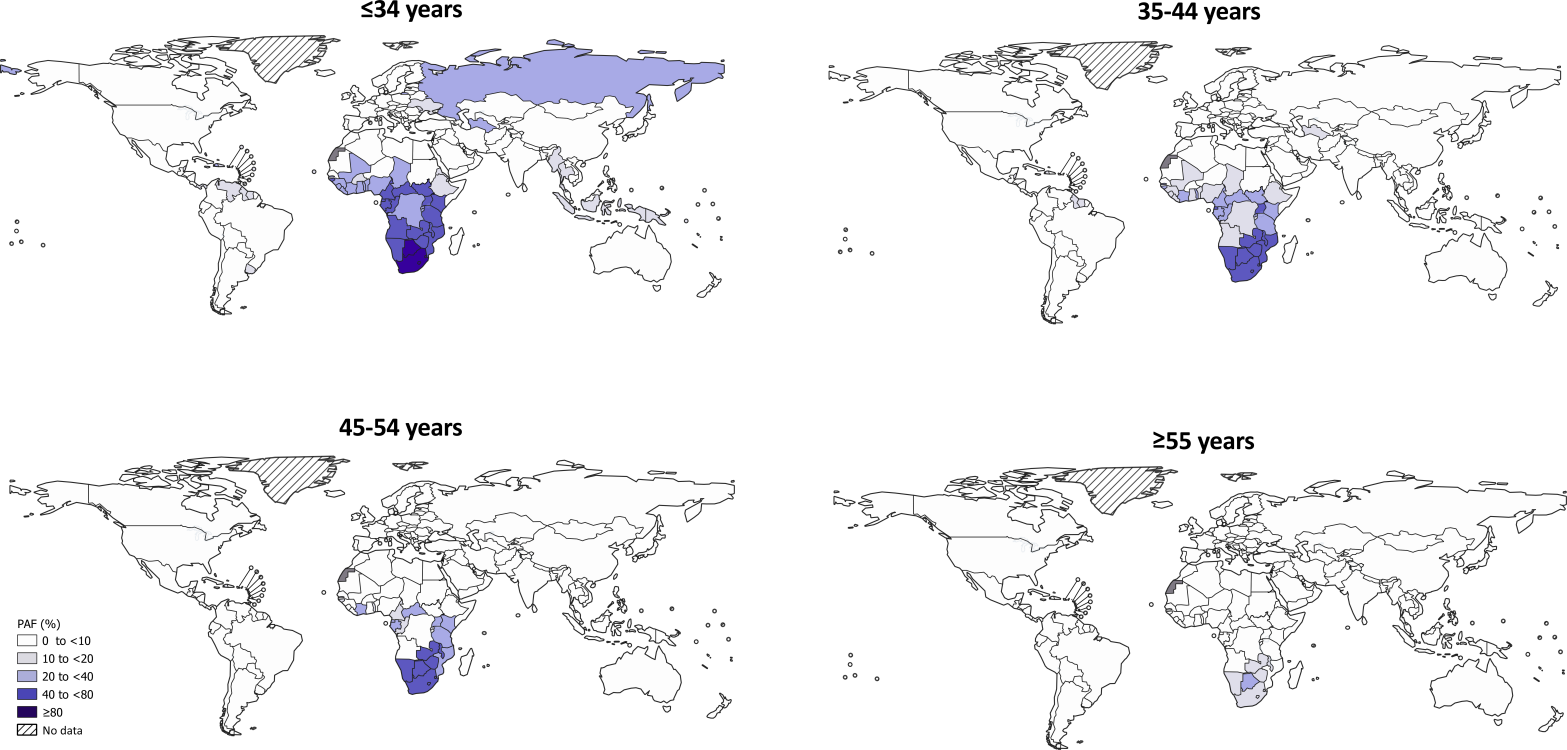
HIV‐attributable fraction in cervical cancer in 2020, by age group. PAF, population‐attributable fraction. The designations used and the presentation of the material in this article do not imply the expression of any opinion whatsoever on the part of WHO and the IARC about the legal status of any country, territory, city, or area, or of its authorities, or concerning the delimitation of its frontiers or boundaries

Geographic and age‐specific patterns of the fraction of cervical cancer among WLHIV and the PAF are mapped in Figures 1 and 2, respectively. Of note, for women diagnosed with cervical cancer at ≤34 years, HIV prevalence was estimated to be higher than 20% in a number of individual countries outside SSA.

### 3.2 Empirical data of HIV prevalence in cervical cancer by age

**FIGURE 3:**
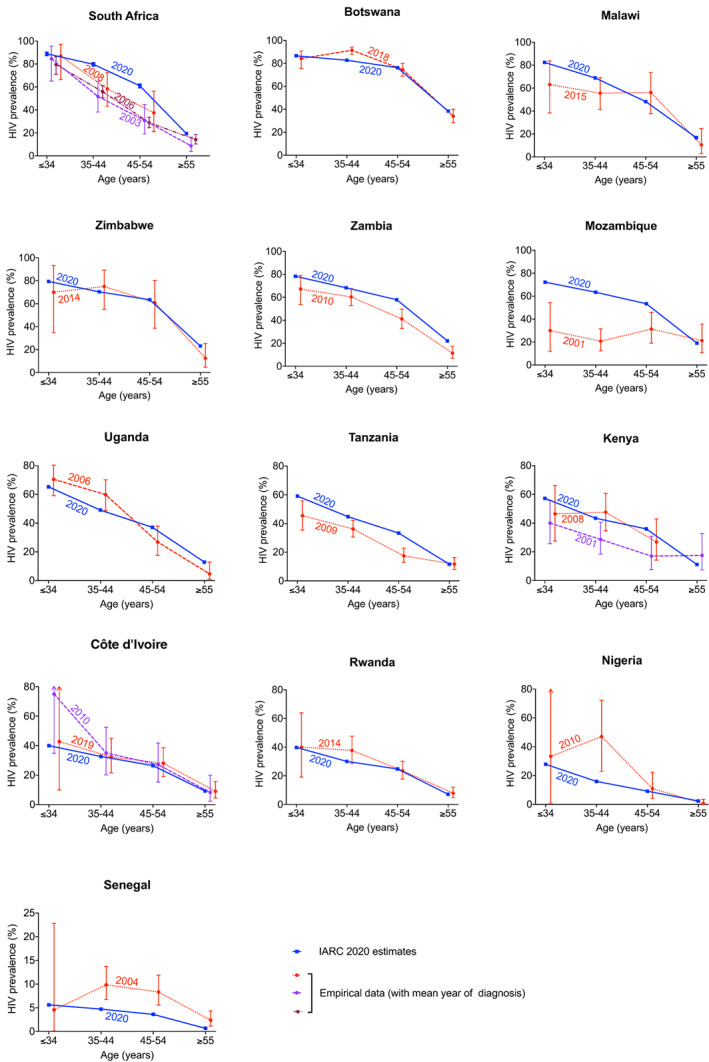
Variation in HIV prevalence in cervical cancer by age: comparison of IARC estimates and empirical data for selected countries. IARC, International Agency for Research on Cancer

As a validation exercise, Figure 3 compares the above‐described IARC age‐specific estimates of HIV prevalence in cervical cancer in 2020 with that of empirical data obtained from 17 studies of consecutively diagnosed cervical cancer cases in 13 SSA countries. Most empirical studies showed strong decreases in HIV prevalence in cervical cancer according to age and were consistent with our 2020 estimates, particularly those studies with a more recent median year of cervical cancer diagnosis. For example, in Botswana, Malawi and Zimbabwe, with mean years of cervical cancer diagnosis of 2018, 2015 and 2014, respectively, and very high HIV prevalence, almost all IARC 2020 age‐specific estimates of HIV prevalence fell within the 95% CI of the age‐specific empirical data. For those SSA countries where empirical studies were more historical (eg, Mozambique and Kenya, mean year of cervical cancer diagnosis = 2001), IARC age‐specific HIV prevalence estimates tended to be higher than those of the empirical data. Finally, in South Africa, age‐specific HIV prevalence in cervical cancer, particularly those diagnosed above 45 years, tended to be higher in series with later mean year of cervical cancer diagnosis.

### 3.3 Absolute burden of invasive cervical cancer cases diagnosed in WLHIV and attributable to HIV

**FIGURE 4:**
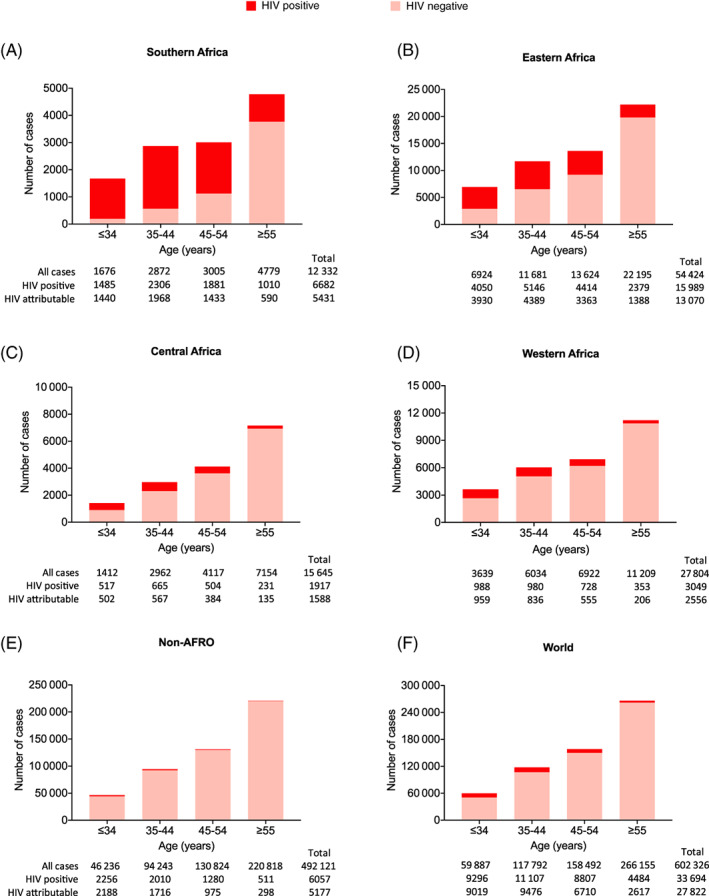
Burden of invasive cervical cancer cases according to HIV status, by world region. Non‐AFRO includes WHO regions: Europe (EURO), South‐East Asia (SEARO), Americas (PAHO), East Mediterranean (EMRO) and Western Pacific (WPRO)

By applying the fractions described in Table 1 to 2020 GLOBOCAN estimates of cervical cancer, 33 694 cases were estimated to be diagnosed in WLHIV globally, and 27 822 of these to be directly attributable to HIV (Figure 4). A vast majority of these cases, 27 637 and 22 645, respectively, were diagnosed in SSA. These included 6682 and 5431 cases in Southern Africa, 15 989 and 13 070 in Eastern Africa, 1917 and 1588 in Central Africa and 3049 and 2556 in Western Africa, respectively (Figure 4). A total of 6059 HIV‐positive and 5178 HIV‐attributable cervical cancer were diagnosed in non‐AFRO regions, outside SSA (Figure 4). By describing numbers of cervical cancer cases according to HIV status and age, Figure 4 highlights the shift in absolute burden of HIV‐positive cervical cancer cases toward younger ages, a phenomenon that is most apparent in Southern Africa. Absolute burden of cervical cancer by HIV attribution status is shown at a country level in Table S1.

### 3.4 Incidence rates of cervical cancer, by HIV attribution status

**FIGURE 5:**
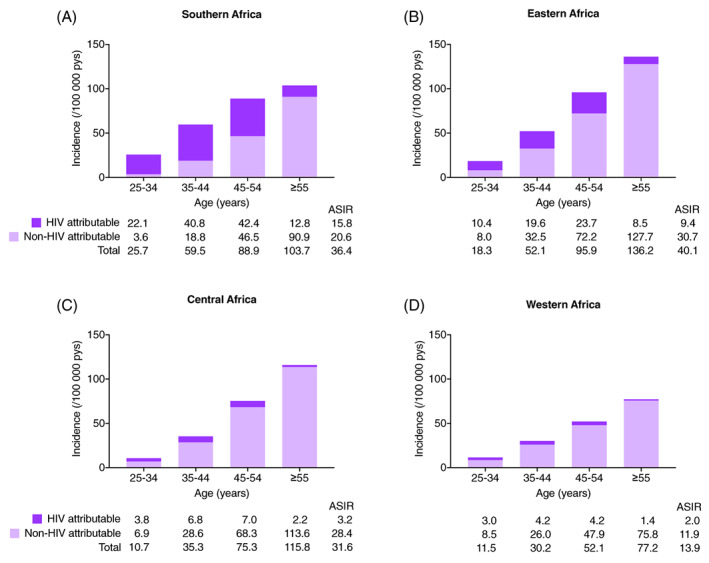
Age‐specific incidence rates of cervical cancer according to HIV‐attribution status, by sub‐Saharan Africa region (not shown for 0‐24 years as negligible [<1 per 100 000 in all regions]). ASIR, age‐standardized incidence rate; pys, person years

Figure 5 describes age‐specific incidence rates of cervical cancer in the four UN African sub‐regions according to HIV‐attribution status, as well as ASIRs. The incidence of non‐HIV‐attributable cervical cancer increased strongly and consistently with age in all regions. ASIRs of non‐HIV‐attributable cervical cancer were highest in Eastern Africa (30.7 cases per 100 000 person years), followed by Central Africa (28.4) and Southern Africa (20.6), and lowest (although still high on a global scale) in Western Africa (11.9) (Figure 5). Age‐specific incidence rates of HIV‐attributable cervical cancer, on the other hand, peaked in age groups 35 to 44 and 45 to 54 years, and ASIRs were highest in Southern Africa (15.8 cases per 100 000 person years), followed by Eastern Africa (9.4), and lowest in Central Africa (3.2) and Western Africa (2.0).

## 4 DISCUSSION

Globally, in 2020, an estimated 6% of new cervical cancer cases were diagnosed among WLHIV, and 5% were estimated to be directly attributable to HIV infection, consistent with our previous estimates for 2018.^2^ However, the age‐specific approach we applied in this effort goes further to demonstrate how the burden of HIV‐attributable cervical cancer falls disproportionately on younger women. This phenomenon was most apparent, both in data obtained from empirical series and in model estimates, in high HIV‐attributable cervical cancer burden settings in SSA. In Southern Africa, for example, where more than half of all incident cervical cancer cases are diagnosed in WLHIV, 63% of HIV‐attributable cervical cancer occurred in women less than 45 years old, compared to only 17% of non‐HIV‐attributable cervical cancer. Improved quantification of cervical cancer burden by age and HIV status can inform appropriate resource allocation in cervical cancer prevention efforts, particularly in SSA.

A number of clinical series from SSA have reported mean age of diagnosis of cervical cancer to be lower in HIV‐infected than HIV‐uninfected women,^13^, ^15^, ^19^, ^20^, ^22^, ^23^, ^24^, ^25^, ^26^, ^27^ most of which provided empirical data to the present effort. However, robust estimates of RR for cervical cancer in WLHIV according to age only became available following the publication of a large IARC‐led case‐control study of HIV and cervical cancer in Rwanda.^4^ Our study was set in an SSA population in the era of widespread access to cART. The age‐specific ORs from our study underpin the current approach and were observed to decrease strongly by age, from OR = 34 (albeit with wide 95% CIs) in women ≤34 years, down to only 2.4 in women aged ≥55 years.

The validity of using age‐specific RRs in our model was confirmed by reproducing the HIV prevalence observed in contemporary cervical cancer cases series, in which HIV prevalence was also observed to decrease strongly by age. Indeed, a model applying the single overall RR (ie, 6) to age‐specific HIV prevalence did not reproduce age‐specific HIV prevalence in these recent cervical cancer series as precisely as an age‐specific RR approach (Figure S1). Interestingly, the age‐specific RR approach was able to reproduce contemporary empirical evidence in settings with broadly different HIV prevalence (eg, Botswana and Côte d'Ivoire), clearly illustrating that RRs are independent of HIV prevalence. Rather, the reason RRs decrease so strongly by age is likely driven by the strong underlying age‐specific differences in cervical cancer risks in HIV‐uninfected women, as well as effects of competing HIV‐related mortality at older ages.

Indeed, temporal fluctuations in competing HIV‐related mortality have had an important historical influence on relative and absolute cervical cancer risk in WLHIV in SSA. In the pre‐cART era of the HIV epidemic in SSA, a moment of severely reduced life expectancy for WLHIV, observed RRs for cervical cancer tended to be non‐significant or relatively weak.^24^, ^28^, ^29^, ^30^, ^31^ Subsequently, in the era of wider access to cART, RRs for cervical cancer in WLHIV in SSA have become increasingly stronger as WLHIV live into the age groups when they are at risk for developing cervical cancer.^4^, ^7^ The RRs reported by Mpunga et al used to inform our estimates were among the highest reported in SSA to date,^2^ likely representing early roll out of cART in Rwanda. At the other extreme, high levels of competing HIV‐related mortality likely explain the much lower HIV prevalence observed in historical pre‐cART empirical series of cervical cancer (eg, Mozambique with mean year of diagnosis of 2001^12^), compared to our recent estimates for the same countries. These extreme changes in life expectancy and cervical cancer risk in WLHIV, over the space of only a few decades, illustrate why recent estimates, whether they be modeled or empirical, are necessary to inform on the current burden of cervical cancer by HIV status in SSA, in the era of widespread access to cART.

Throughout this work, we present two measures of the burden of HIV‐associated cervical cancer, namely that of cervical cancer diagnosed among WLHIV (equating to HIV prevalence among cases of cervical cancer), and that of cervical cancer attributable to HIV (equating to the PAF). Both these measures are important for informing different aspects of cervical cancer prevention.

The burden of HIV‐attributable cervical cancer gives a measure of cancer cases directly due to HIV, that is, the number or proportion of cases that could theoretically be avoided if HIV was removed from the population. This measure, thus, brings into focus the importance of reducing HIV transmission with respect to the goal of elimination of cervical cancer in SSA.^32^, ^33^ Earlier diagnosis of HIV infection and timelier initiation of cART will also contribute to reducing the HIV‐attributable cervical cancer burden, as cART is known to significantly lower the risk of cervical cancer among WLHIV.^34^ Of note, since HIV and HPV share a common transmission route (sexual contact), there is a potential for unmeasured confounding to translate into overestimated RRs, and thereby PAFs, particularly in settings with the highest prevalence of HIV infection. Nevertheless, without any possibility of controlling this interaction, these estimates provide the best current approximation of the excess cervical cancer burden directly attributable to HIV (and that would not exist in the absence of the HIV epidemic).

Estimates of the proportion of cervical cancer cases diagnosed among WLHIV, on the other hand, are unaffected by the above methodological consideration. Furthermore, they can be directly validated against empirically observed evidence, as illustrated in the current exercise. This measure can be used to understand the extent to which burden of disease falls on WLHIV, irrespective of direct causality, and is, thus, particularly relevant for informing the design, and potential impact, of cervical cancer prevention services targeted to WLHIV.

In recognition of the increased risk of cervical cancer in WLHIV, it is widely recommended for cervical screening of WLHIV to start at an earlier age, and for subsequent screening intervals to be shorter for WLHIV than for the general female population.^35^ In SSA, and some other settings without population‐level cervical cancer screening programs, this has led to several cervical cancer screening initiatives that primarily target WLHIV, most notably those funded by PEPFAR.^36^ This approach can be facilitated by WLHIV undergoing regular follow‐up in the healthcare system. Furthermore, investment in new screening infrastructures for WLHIV can be a catalyst for subsequent expansion of these services to the wider HIV‐uninfected population.^37^ Thus, age‐specific burden of cervical cancer in WLHIV vs that in HIV‐uninfected women at a population level can help inform the appropriate lower age limit of WLHIV‐targeted cervical cancer screening, as well as assess the relative impact of a targeted approach for WLHIV vs that of a more general population program.^32^, ^38^

The WHO global strategy to eliminate cervical cancer is ultimately underpinned by widespread implementation of HPV vaccination programs. Vaccination prior to sexual activity is expected to prevent cervical cancer in vaccinated cohorts, but this impact will not occur for a number of decades and will first be seen on cervical cancer burden at youngest ages.^39^ Vaccine impact should be witnessed irrespective of HIV status, given that HIV is not a direct carcinogen, but rather acts via impaired immunity to worsen the carcinogenic effect of HR HPV. Hence, increasing HPV vaccination coverage in countries with a high burden of cervical cancer, particularly those with a double burden of HPV and HIV, remains a critical priority. This is especially the case as these same high burden countries are often those where population‐wide cervical screening coverage is low.^40^

Of note, all ages combined, overall burden estimates of HIV‐associated cervical cancer at a country, regional and global level for 2020 are materially unchanged from those of 2018.^2^ For example, the approximate number of cervical cancers diagnosed in WLHIV globally was estimated at 33 000 in 2018 and 34 000 in 2020 and 28 000 for WHO/AFRO in both years. Indeed, the nature of GLOBOCAN data on cervical cancer burden (and, to a certain extent also that of UNAIDS HIV prevalence data) means that estimates from subsequent editions should not be considered as time trends, but rather as best possible current estimates given continual data improvements. Of note, many SSA settings do not have population‐based cancer registries, and GLOBOCAN estimates are often derived from hospital cancer series, mortality data, or are based on data from neighboring countries. As in previous estimates for 2018,^2^ we show ASIR of cervical cancer by HIV‐attribution status and additionally show, for the first time, age‐specific incidence rates by HIV‐attribution status. These measures are useful to compare the contribution of HIV to population‐level cervical cancer burden across different SSA populations, in a standardized format.

It should be noted that HIV‐attributable cancer incidence among all women (as shown here) is not the same as cervical cancer incidence in WLHIV. Few data exist on age‐specific cervical cancer incidence in WLHIV in SSA. However, some preliminary data have been recently reported by a large linkage study in South Africa^41^ describing, as expected, strongly increasing cervical cancer incidence in WLHIV by age: from 29 cases per 100 000 persons years for WLHIV aged 20 to 29 years up to 242 cases per 100 000 person years at 60 to 69 years.^41^ Similar measures are not easily calculable from our approach, due to difficulties in establishing person years by HIV status at a population level.

The fact that there were no relevant empirical cervical cancer series against which to validate our HIV estimates outside SSA is a limitation of this analysis. In the United States, however, studies based on linkage of HIV/AIDS and cancer registries have also shown the contribution of HIV to cervical cancer to be shifted toward younger ages. In a setting of low HIV prevalence and widespread cervical screening, the proportion of cervical cancer in 1980 to 2007 in the United States, diagnosed among women with AIDS, has been estimated at 0.8%, 0.6% and 0.04% at ages 0 to 29, 30 to 59 and ≥60 years, respectively.^42^ These estimates compare to 2.8%, 1.7%, 1.3% and 0.3% for ≤34, 35 to 44, 45 to 54 and ≥55 years diagnosed among WLHIV in the United States in our 2020 estimates.

In conclusion, the burden of cervical cancer associated with HIV is strongly shifted toward women at younger ages and has been changing with the evolution of the HIV epidemic in SSA. Thus, locally relevant data on the age‐specific contribution of HIV to cervical cancer should be used to better design prevention programs, particularly in settings in SSA hit by a double burden of HPV and HIV. To keep evidence up to date, SSA countries should be encouraged to document HIV status and cART use in clinical cervical cancer series. This should be feasible given that cervical cancer is an AIDS‐defining condition, and that HIV‐testing is becoming increasingly widespread. These data will be highly informative for monitoring progress toward the WHO cervical cancer elimination goal in SSA.

## CONFLICT OF INTEREST

The authors declare no conflicts of interest.

## Supporting information


**TABLE S1** Absolute burden of invasive cervical cancer cases according to HIV status at country level in 2020

**FIGURE S1** Variations in HIV prevalence in cervical cancer by age: comparison between estimates using age‐specific relative risk, estimates using overall relative risk and empirical data

## Data Availability

The data that support the findings of our study derive from several sources. Publicly available sources are: cancer incidence in five continents volume XI (http://ci5.iar.fr/) and Globocan 2020 (Global Cancer Observatory https://gco.iarc.fr/today/home). For included studies, please refer to cited published references. Further information is available from the corresponding author upon request.
