# Supplementary material for: Age‐specific burden of cervical cancer associated with HIV: A global analysis with a focus on sub‐Saharan Africa
Source: Int J Cancer. 2021 Oct 19;150(5):761–72. doi: 10.1002/ijc.33841 (PMC8732304; doi:10.1002/ijc.33841)
Supplement: Supplementary file 1 — TABLE S1 Absolute burden of invasive cervical cancer cases according to HIV status at country level in 2020 [file IJC-150-761-s002.pdf]

Supporting information

Age-specific burden of cervical cancer associated with HIV: a global analysis with a focus on sub-Saharan Africa

Ahmadaye Ibrahim Khalil, Tharcisse Mpunga, Feixue Wei, Iacopo Baussano, Catherine de Martel, Freddie Bray, Dominik Stelzle, Scott Dryden-Peterson, Antoine Jaquet, Marie-Josèphe Horner, Olutosin A Awolude, Mario Jesus Trejo, Washington Mudini, Amr S. Soliman, Mazvita Sengayi-Muchengeti, Anna E. Coghill, Matthys C van Aardt, Hugo De Vuyst, Stephen E Hawes, Nathalie Broutet, Shona Dalal, Gary M Clifford.

Table of contents

TABLE S1 Absolute burden of invasive cervical cancer cases according to HIV status at country-level in 2020 (provided as separate excel file)

FIGURE S1 Variations in HIV prevalence in cervical cancer by age: comparison between estimates using age-specific relative risk, estimates using overall relative risk and empirical data 2

FIGURE S1 Variations in HIV prevalence in cervical cancer by age: comparison between estimates using age-specific relative risk, estimates using overall relative risk and empirical data

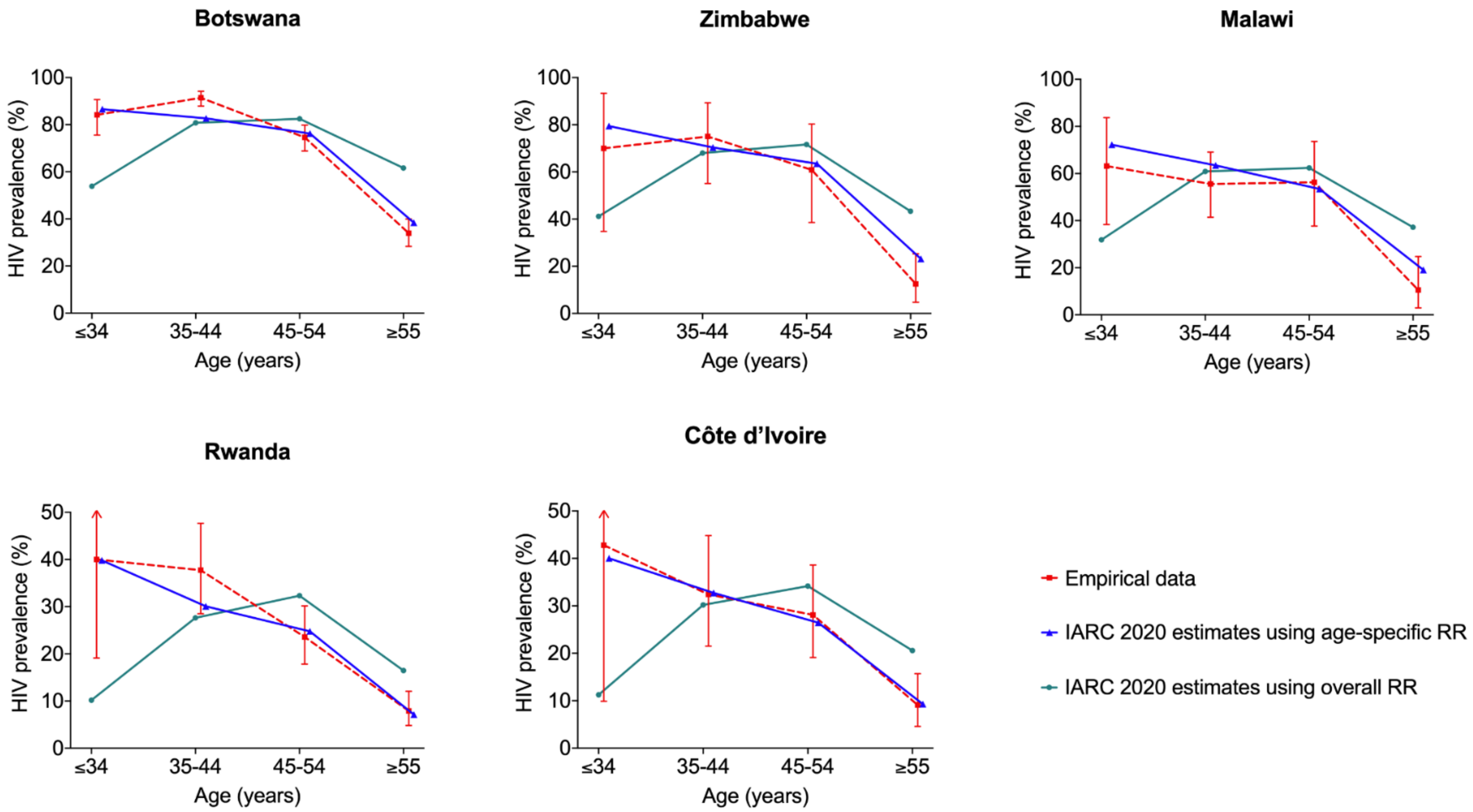

For Cote d'Ivoire, empirical data series with mean year of diagnosis 2018 is shown.
IARC, International Agency for Research on Cancer; RR, relative risk.
